# Restrictive vs liberal red blood cell transfusion strategies in patients with acute myocardial infarction and anemia: Rationale and design of the REALITY trial

**DOI:** 10.1002/clc.23453

**Published:** 2021-01-06

**Authors:** Gregory Ducrocq, Gonzalo Calvo, José Ramón González‐Juanatey, Isabelle Durand‐Zaleski, Cristina Avendano‐Sola, Etienne Puymirat, Gilles Lemesle, Joan Albert Arnaiz, Manuel Martínez‐Sellés, Alexandra Rousseau, Marine Cachanado, Eric Vicaut, Johanne Silvain, Carma Karam, Nicolas Danchin, Tabassome Simon, Philippe Gabriel Steg, REALITY investigators

**Affiliations:** ^1^ Université de Paris, AP‐HP French Alliance for Cardiovascular Trials (FACT), INSERM U1148 Paris France; ^2^ Àrea del Medicament Hospital Clínic of Barcelona University of Barcelona Barcelona Spain; ^3^ Cardiology Department, University Hospital, IDIS, CIBERCV University of Santiago de Compostela Santiago de Compostela Spain; ^4^ AP‐HP Health Economics Research Unit Hotel Dieu Hospital, INSERM UMR 1153 CRESS Paris France; ^5^ Clinical Pharmacology Service Hospital Universitario Puerta de Hierro‐Majadahonda Madrid Spain; ^6^ Hôpital Européen Georges Pompidou, AP‐HP French Alliance for Cardiovascular Trials (FACT), and Université de Paris Paris France; ^7^ Institut Cœur Poumon, Centre Hospitalier Universitaire de Lille, Faculté de Médecine de Lille Université de Lille, Institut Pasteur de Lille, Inserm U1011, F‐59000 Lille, France; French Alliance for Cardiovascular Trials (FACT) Paris France; ^8^ Clinical Trials Unit, Clinical Pharmacology Department Hospital Clinic Barcelona Spain; ^9^ Servicio de Cardiología, Hospital Universitario Gregorio Marañón, CIBERCV; Universidad Europea Universidad Complutense Madrid Spain; ^10^ Clinical Research Platform (URCEST‐CRB‐CRCEST), AP‐HP Hôpital Saint Antoine Paris France; ^11^ AP‐HP, Department of Biostatistics Université Paris‐Diderot, Sorbonne‐Paris Cité, Fernand Widal Hospital France; ^12^ Sorbonne Université, ACTION Study Group Institut de Cardiologie, Hôpital Pitié‐Salpêtrière, AP‐HP, INSERM UMRS 1166 Paris France; ^13^ Cardiology Department, Ambroise Paré Hospital, AP‐HP, Boulogne University of Versailles‐Saint Quentin en Yvelines Boulogne‐Billancourt France; ^14^ Department of Clinical Pharmacology‐Clinical Research Platform (URCEST‐CRB‐CRCEST), AP‐HP, Hôpital Saint Antoine, French Alliance for Cardiovascular Trials (FACT) Sorbonne‐Université Paris France; ^15^ Royal Brompton Hospital Imperial College London UK

**Keywords:** acute myocardial infarction, anemia, cost effectiveness, transfusion

## Abstract

**Background:**

Anemia is common in patients with acute myocardial infarction (AMI), and is an independent predictor of mortality. The optimal transfusion strategy in these patients is unclear.

**Hypothesis:**

We hypothesized that a “restrictive” transfusion strategy (triggered by hemoglobin ≤8 g/dL) is clinically noninferior to a “liberal” transfusion strategy (triggered by hemoglobin ≤10 g/dL), but is less costly.

**Methods:**

REALITY is an international, randomized, multicenter, open‐label trial comparing a restrictive vs a liberal transfusion strategy in patients with AMI and anemia. The primary outcome is the incremental cost‐effectiveness ratio (ICER) at 30 days, using the primary composite clinical outcome of major adverse cardiovascular events (MACE; comprising all‐cause death, nonfatal stroke, nonfatal recurrent myocardial infarction, or emergency revascularization prompted by ischemia) as the effectiveness criterion. Secondary outcomes include the ICER at 1 year, and MACE (and its components) at 30 days and at 1 year.

**Results:**

The trial aimed to enroll 630 patients. Based on estimated event rates of 11% in the restrictive group and 15% in the liberal group, this number will provide 80% power to demonstrate clinical noninferiority of the restrictive group, with a noninferiority margin corresponding to a relative risk equal to 1.25. The sample size will also provide 80% power to show the cost‐effectiveness of the restrictive strategy at a threshold of €50 000 per quality‐adjusted life year.

**Conclusions:**

REALITY will provide important guidance on the management of patients with AMI and anemia.

## 1 INTRODUCTION

Anemia is common in patients with acute myocardial infarction (AMI),^1^ and is an independent predictor of cardiac events and increased mortality.^1^, ^2^, ^3^, ^4^ The association with increased mortality is strong, with a relative increase in mortality exceeding 20% for each 1 g/dL decrement in hemoglobin below 14 g/dL.^2^ The antiplatelet and anticoagulant drugs used for the treatment of patients with AMI increase the risk of bleeding, which in turn increases the risk of ischemia and death. Whether this risk can be overcome by transfusion is debated.

Clinical data on the effects of blood transfusion in patients with AMI are observational^5^, ^6^ or based on small, underpowered randomized trials.^7^, ^8^ Previous results have been inconsistent, which prompted a call for large randomized trials.^9^, ^10^ As a consequence, only low levels of evidence are available to guide international guidelines on blood transfusion in patients with AMI and anemia, which are somewhat inconsistent.^11^, ^12^, ^13^

Randomized trials comparing transfusion strategies in patients undergoing noncardiac^14^ or cardiac surgery,^15^, ^16^ and in patients with upper gastrointestinal bleeding,^17^ have generally found benefit from a restrictive transfusion strategy. However, these trials excluded patients with AMI. In the specific context of acute myocardial ischemia, the effect of transfusion strategies may differ markedly from what is observed in other settings. Apart from the clinical benefit of either strategy, other issues should be considered: blood is a costly and scarce resource; transfusion can be associated with adverse events (infectious and noninfectious); and transfusion mobilizes substantial resources and healthcare professional time, such that even if a restrictive strategy were noninferior to a liberal transfusion strategy, there may be value in preferring the former.

Given the high prevalence of anemia, its strong association with death and adverse cardiac outcomes, the cost implications of transfusion, and the lack of high‐quality data to guide clinical practice in patients with AMI and anemia, we conducted an international, randomized clinical trial comparing a “restrictive” (triggered by hemoglobin ≤8 g/dL) vs a “liberal” (triggered by hemoglobin ≤10 g/dL) red blood cell (RBC) transfusion strategy in patients with AMI and anemia (defined as 7 g/dL < hemoglobin ≤10 g/dL). We hypothesized that a restrictive transfusion strategy would be clinically noninferior to a liberal transfusion strategy but more cost effective.

## 2 METHODS

### 2.1 Study organization

The REstrictive And LIberal Transfusion strategies in patients with AMI (REALITY) trial (ClinicalTrials.gov Identifier: NCT02648113) is a European (France and Spain), randomized, multicenter, open‐label trial designed by an independent academic group (French Alliance for Cardiovascular Trials [FACT]) and sponsored by Assistance Publique − Hôpitaux de Paris in France and by Sociedad Espanola de Farmacologia Clinica in Spain. The study was conducted in 35 clinical sites (26 in France, 9 in Spain).

The steering committee is responsible for the medical, scientific, and operational conduct of the study, and vouches for the integrity of the data analysis and the reporting of the results. The study adheres fully to the ethical principles of the Declaration of Helsinki. The duration of participation for each patient was 1 year.

### 2.2 Study population

Patients ≥18 years with AMI and anemia who met the inclusion criteria and none of the exclusion criteria (Table 1) were enrolled at 35 sites between 23 March 23 2016 and 10 October 2019. We aimed to enroll 630 patients in the main study and 100 in the ancillary study. Inclusions in the main study could be extended to attain this goal. All patients provided written informed consent.

**TABLE 1:** Inclusion and exclusion criteria in the REALITY trial

Inclusion criteria	Exclusion criteria^a^
Age ≥ 18 y Recent AMI, with or without ST‐segment elevation, with a combination of ischemic symptoms occurring in the past 48 h before the myocardial infarction‐related admission and elevation of biomarkers of myocardial injury (troponin) Anemia (7 g/dL < hemoglobin ≤10 g/dL) at any time during the index hospital admission for myocardial infarction Written informed consent Coverage for medical insurance	Shock (SBP <90 mmHg with clinical signs of low output or requiring inotropic agents) Myocardial infarction occurring post‐PCI or post‐CABG (ie, type IV or V AMI according to the 2012 Universal definition of myocardial infarction^18^) Life‐threatening or massive ongoing bleeding (as judged by the investigator) Any blood transfusion in the previous 30 d Known malignant hematologic disease^a^

Abbreviations: AMI, acute myocardial infarction; CABG, coronary artery bypass grafting; PCI, percutaneous coronary intervention; SBP, systolic blood pressure.

^a^ Sickle cell disease, thalassemia, and anemia due to chronic renal failure (even under erythropoietin) are not exclusion criteria.

### 2.3 Study outcomes

The primary outcome is the incremental cost‐effectiveness ratio (ICER) at 30 days, using major adverse cardiovascular events (MACE) (composite of all‐cause death, nonfatal stroke, nonfatal recurrent myocardial infarction, or emergency revascularization prompted by ischemia) as the effectiveness criterion. The primary and various secondary outcomes are listed in Table 2.

**TABLE 2:** Outcomes of the REALITY trial

Outcomes	Details
Primary clinical effectiveness composite outcome	
Clinical noninferiority at 30 d	MACE^a^ at 30 days
Primary endpoint	
ICER at 30 d	ICER at 30 days, using the 30 day composite endpoint of MACE^a^ as the effectiveness criterion
Secondary endpoints	
Exploratory outcomes at 30 d	All the components^a^ of the composite clinical outcome will be analyzed separately
Component ICERs at 30 d	ICERs for each individual component of the primary endpoint will be estimated separately
Clinical noninferiority at 1 y	MACE^a^ at 1 y All the components^a^ of this composite clinical outcome will also be analyzed separately as secondary endpoints
ICER at 1 y	ICER at 1 year, using the 1 y composite endpoint of MACE^a^ as the effectiveness criterion
ICUR at 1 y	ICUR at 1 year using QALY at 1 y

Abbreviations: ICER, incremental cost‐effectiveness ratio; ICUR, incremental cost‐utility ratio; MACE, major adverse cardiovascular events.

^a^ All‐cause death, nonfatal stroke, nonfatal recurrent myocardial infarction, or emergency revascularization prompted by ischemia. All components of the composite outcome will also be analyzed separately.

All clinical outcomes (deaths, including cause of death, AMIs, strokes, ischemia‐driven emergency revascularizations, and heart failures) will be reviewed and adjudicated by a CEC. The CEC will be given all relevant clinical and biochemical information and will be blinded to the randomization assignment. In addition, AMIs will be categorized by the CEC according to the Third Universal Definition.^18^ Each triggered event will be reviewed by two CEC members. In case of discrepancy between the two adjudicators, an ad hoc committee with at least three CEC members will reach a final decision based on consensus.

Safety outcomes of special interest, which will be monitored during the index hospitalization, include hemolysis, infections, multiorgan system dysfunction, acute lung injury, acute heart failure, acute renal failure, and severe allergic reactions.

### 2.4 Health‐economic analysis

A cost‐effectiveness primary outcome (30 day ICER) was chosen due to its frequent use in cardiology and the availability of benchmarks for the cost‐effectiveness results.^19^, ^20^ The BASKET trial has provided a benchmark for the acceptable cost per MACE averted with an ICER of 64 732 euros to prevent one major adverse cardiac event.^21^ The secondary health outcome for the cost‐utility analyses is quality‐adjusted life years (QALYs) at 1 year. These are calculated for both groups using health‐related quality of life (HRQoL) scores from the EuroQoL five dimensions (EQ‐5D) questionnaire and converted to utility scores using country weights.^22^, ^23^

The cost‐effectiveness analysis of the costs and outcomes for patients in the restrictive and liberal transfusion groups will be conducted from the perspectives of both the French and Spanish governments (providing a healthcare system perspective for both countries). This perspective allows comparisons with the results of other economic evaluations in cardiology. The economic evaluation will be based on the entire population of patients in the trial. Unit costs are undiscounted and are presented in Tables S1 and S2. Resources will be collected prospectively at the patient level. Prespecified subgroup analyses by country are necessary due to potential differences in unit costs and resource utilization.^24^

### 2.5 Transfusion strategies and randomization

**FIGURE 1:**
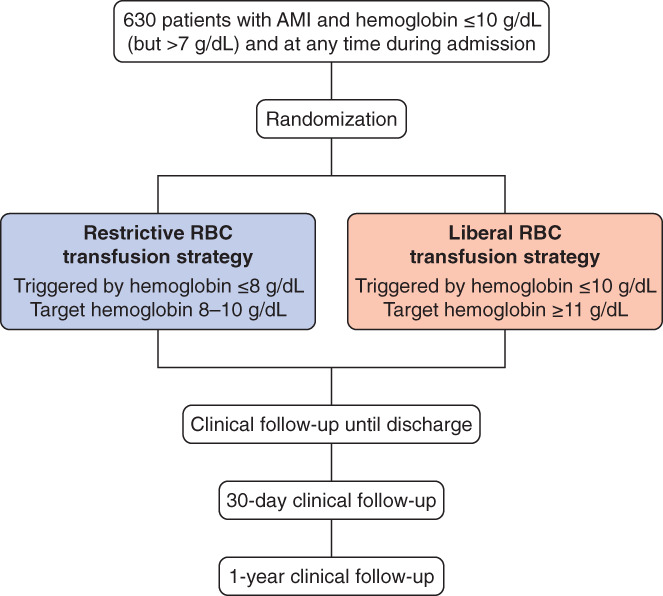
REALITY trial flow chart. AMI, acute myocardial infarction; RBC, red blood cell

A centralized, blocked randomization list with blocks of varying size, stratified by center, was used. The investigators at each site obtained the randomized strategy allocation using the internet (CleanWeb, Telemedecine Technologies, S.A.S). CleanWeb assigned each patient a unique randomization number that corresponded to one of the two transfusion strategies (Figure 1):

- A restrictive transfusion strategy, where transfusions are withheld unless hemoglobin is ≤8 g/dL, with a target hemoglobin of 8‐10 g/dL;
- A liberal transfusion strategy, where transfusions are allowed as soon as hemoglobin is ≤10 g/dL, with a target hemoglobin of ≥11 g/dL.

The strategies should be maintained until discharge from hospital or for 30 days, whichever comes first. Special steps are being taken to ensure adherence to the strategy assigned by randomization (particularly if patients are moved to another hospital department): patients receive colored bracelets delineating the transfusion strategy, colored reminder stickers are included in the patients' files, and posters are displayed in the intensive care units. Of note, all medications are left to the discretion of the treating physician and are not dictated by the study protocol.

### 2.6 Follow‐up

After randomization, the following data are being collected in each group: number of RBC units, type of RBC (autologous or homologous), duration of RBC conservation, and associated transfusion of non‐RBC blood products (platelets, coagulation factors, etc.). The collection of outcome data is being performed by the investigator at discharge or 30 days, whichever occurs first. After discharge, patient follow‐up is scheduled at Day 30 (±5 days) and follow‐up data are collected by the investigator, either by direct contact (if the patient is still hospitalized) or by a visit, phone call, or mail. The EQ‐5D HRQoL questionnaire is completed by the patients.

At 6 months (±30 days) and 1 year (±30 days), patients are contacted to collect clinical event and HRQoL (EQ‐5D questionnaire) data.

### 2.7 Statistical methods

#### 2.7.1 Power and sample size considerations

There is no established clinical superiority of either transfusion strategy at this stage. We hypothesized that a restrictive transfusion strategy would be clinically noninferior to a liberal transfusion strategy but less costly. As our hypotheses are clinical noninferiority but superior cost‐effectiveness of the restrictive transfusion strategy, we based our power calculations on clinical grounds. We anticipated MACE rates of 11% in the restrictive transfusion strategy and 15% in the liberal transfusion strategy, based on observations from the nationwide French registry of acute ST‐Elevation or non‐ST‐elevation myocardial infarction (FAST‐MI) program.^25^ Based on these hypotheses, a sample size of 300 patients per group would provide 80% power to demonstrate noninferiority of the restrictive group, with a noninferiority margin corresponding to a relative risk equal to 1.25, using a confidence interval (CI) method with a 97.5% one‐sided CI. With a conservative hypothesis of 5% of patients with major protocol violations, a total of 630 patients (315 in each arm) are required for the trial to be adequately powered for both intention‐to‐treat (ITT) and per‐protocol (PP) analyses. Using the formula for sample size calculation in economic evaluation, we estimated that this sample size would allow us to show cost‐effectiveness at a threshold of €50 000, a power of 80%, and a significance level of 5%, a €1000 difference in costs and 0.15 difference in QALYs.^26^

$$n=\frac{2\;{\left(z_{\propto }+z_{\beta }\right)}^{2}\left({\mathrm{sd}}_{c}^{2}+{\left(\mathrm{Ws}d_{q}\right)}^{2}-\left(2\mathrm{W\rho s}d_{c}sd_{q}\right)\right)}{{\left(W∆Q-∆C\right)}^{2}}.$$

where *C* and *Q* represent costs and utilities, *W* willingness to pay, and *ρ* the correlation between costs and quality of life (set at −0.1).

#### 2.7.2 Statistical analyses

Analyses will be performed in both the ITT and PP cohorts. The ITT population is defined as all randomized patients who have signed an informed consent form. In case of consent withdrawal, only data collected before withdrawal will be used. All patients will be analyzed in their randomized arm regardless of the strategy used during the trial. The PP population is defined as all patients randomized and treated without major protocol violations or deviations. Predefined major protocol violations/deviations are: (1) violation of the selection criteria; (2) receipt of the alternative transfusion strategy (apart from in the situations listed below); (3) missing data for the primary efficacy outcomes; (4) inclusion in another interventional study; and (5) major protocol deviation identified during a blinded data review before database lock. Crossover from the restrictive to the liberal strategy will be allowed, and thus not considered a major protocol deviation (so these patients will be included in the PP analysis), in the following documented cases: massive overt active bleeding, presumed important fall in hemoglobin level and no time to wait for hemoglobin testing, or shock occurring after randomization. All statistical analyses will be performed using SAS 9.4 software.

#### 2.7.3 Cost‐effectiveness analysis

A joint comparison of costs and MACE will be performed by nonparametric bootstrapping with 1000 re‐samples. A distribution will be attributed to each variable according to accepted practice: normal distribution for MACE and log normal or Beta for costs. The results of the bootstrap replications will be presented on a cost‐effectiveness plane. In addition to the cost‐effectiveness plane, acceptability curves for French and Spanish values will be plotted.

#### 2.7.4 Cost‐utility analysis

The cost‐utility analysis will be performed with French and Spanish utility values estimated from EQ‐5D scores.

#### 2.7.5 Primary clinical outcome analysis

Noninferiority will be considered to have been demonstrated if the upper value of the 97.5% one‐sided CI of the relative risk is inferior to the noninferiority margin equal to 1.25, in both the PP and ITT populations. If noninferiority of the restrictive strategy is established, sequential testing of superiority of the restrictive strategy will be accepted if the upper value of the 97.5% one‐sided CI of the relative risk is inferior to one.

All individual components of the primary outcome will be analyzed by Chi‐square or Fisher's exact tests. As these analyses will be exploratory, no adjustment of the alpha value for multiplicity will be made.

Times to death and MACE during 1‐year follow‐up will be described by Kaplan‐Meier curves and tested by log‐rank test. The reference date is defined as the randomization date. All patients will be censored at the time of the last observation.

All safety endpoints will be analyzed by Chi‐square or Fisher's exact tests.

### 2.8 Subgroup analyses

Interactions between outcomes and prespecified subgroups will be analyzed according to the following variables: age, sex, body weight, diabetes mellitus, smoking status, hypertension, dyslipidemia, Killip class, type of AMI (ST‐segment elevation myocardial infarction [STEMI] or non‐STEMI), renal function at baseline, use of revascularization for acute coronary syndrome (ACS), hemoglobin at randomization, and presence or absence of overt bleeding before randomization.

### 2.9 Ancillary biological substudy

In France, an ancillary study (Impact of Transfusion of RBC on platelet activation, aggregation, and Systemic biomarkers with clinical Follow‐Up Studied in patients with anemia and myocardial infarction of the REALITY trial; TRANSFUSION‐REALITY) is being performed. The aim is to evaluate the effect of RBC transfusion on platelet activation, aggregation, and systemic biomarkers of thrombosis and inflammation in the REALITY trial in a series of biological measurements: baseline (ie, at randomization; before the first RBC transfusion [T0]), after RBC transfusion (on the day after randomization and/or transfusion according to the group [T1]), and at discharge from the hospital (T2).

The primary outcome is a comparison, between the two groups, of the variation in the level of platelet reactivity using vasodilator‐stimulated phosphoprotein (VASP) between T0 and T1. Up to 100 patients will be included, which will give 85% power with a 15% absolute difference in platelet reactivity. Secondary objectives will be to explore the effect of transfusion on platelet aggregation (using the VerifyNow test), thrombosis biomarkers (CD40 ligand, P‐selectin, plasminogen activator inhibitor‐1, von Willebrand factor, and glycoprotein 6), inflammation biomarkers (tumor necrosis factor alpha, interleukin‐6 and interleukin‐10, and high‐sensitivity C‐reactive protein), and interaction with leukocytes (neutrophil extracellular traps).

### 2.10 Funding

The study is funded via a grant from the Programme de Recherche Médico‐Economique (PRME) from the French Ministry of Health, and a grant from the Instituto de Salud Carlos III (Spanish Ministry of Economy and Competitiveness), Grant n° PI15/01543.

## 3 DISCUSSION

The primary hypothesis of the REALITY trial (NCT02648113) is that a restrictive transfusion strategy (triggered by hemoglobin ≤8 g/dL) will be clinically noninferior to a liberal transfusion strategy (triggered by hemoglobin ≤10 g/dL), but will be less costly.

Physiopathologic considerations suggest that the effect of RBC transfusion may differ between patients with vs without AMI. In theory, transfusion should increase oxygen delivery to the myocardium. However, recent data suggest that oxygen delivery is not increased in patients receiving transfusions, that RBCs are rapidly depleted of nitric oxide during storage, and that, conversely, transfusion may increase platelet activation and aggregation.^27^ These consequences appear potentially even more deleterious in patients with cardiovascular disease.^28^ RBC transfusion can also be associated with various noncardiac complications such as transfusion‐associated infections, acute lung injury, and circulatory overload.^29^ All of these potential complications will be carefully monitored in the REALITY trial.

The issue of a restrictive vs a liberal transfusion strategy is not only relevant in cardiology, but is a common clinical problem encountered in many other settings.^30^ A randomized trial conducted in patients with acute upper gastrointestinal bleeding in Spain showed that a restrictive vs liberal (when hemoglobin is <7 vs <9 g/dL) transfusion strategy was associated with improved 6‐week survival (95% vs 91%; *P* = .02).^17^ However, patients with ischemic heart disease were excluded from enrollment.^17^ A meta‐analysis of randomized trials comparing restrictive vs liberal strategies and including more than 3000 patients with cardiovascular disease^14^ (not only patients with coronary artery disease) found that the risk of ACS was increased in patients managed with the restrictive strategy. However, the studies included in this meta‐analysis included patients in various contexts (eg, hip surgery), which is quite different from the scenario of patients with recent AMI and anemia.

In the field of cardiac surgery, two randomized trials did not find improved outcomes with either restrictive or liberal strategies.^15^, ^16^ However, a meta‐analysis of randomized trials in cardiac surgery showed a trend towards benefit of a liberal transfusion strategy, whereas in observational studies, transfusion (vs no transfusion) was associated with higher mortality.^31^ In critical care patients, a randomized trial found that a liberal transfusion strategy (transfusion started when hemoglobin was <9 g/dL) had no significant benefit compared to a restrictive strategy (transfusion allowed when hemoglobin was <7 g/dL) in terms of 30‐day death (23.3% vs 18.7%; *P* = .11).^32^ However, patients with ischemic heart disease were the only subgroup where the restrictive strategy had a numerically (although not statistically significant) lower absolute 30‐day survival rate (21% vs 26%; *P* = .38),^33^ suggesting that this group may respond differently to transfusion compared with other patients. A similar signal was observed in a meta‐analysis of trials including critically ill patients randomized to restrictive vs liberal strategies, which found a numerical increase in death in the subgroup of patients with AMI treated with a restrictive strategy.^34^

Clinical data regarding transfusions in AMI are mainly observational and yield inconsistent results. An analysis of the Controlled Abciximab and Device Investigation to Lower Late Angioplasty Complications (CADILLAC) trial found that transfusion was independently associated with 30‐day and 1‐year death, and concluded that transfusion may be harmful after primary percutaneous coronary intervention.^35^ Similarly, a pooled analysis of three international ACS trials found that blood transfusion was associated with higher death after adjustment for confounding factors.^36^ Conversely, a retrospective analysis of nearly 80 000 elderly patients hospitalized with AMI reported lower 30 day death in those with hematocrit <33% who received transfusions vs those who did not (adjusted odds ratio [OR] 0.69, 95% CI 0.53‐0.89).^5^ A meta‐analysis (>200 000 patients) reported a higher risk of death and recurrent myocardial infarction in patients with AMI who received transfusions (OR 2.91, 95% CI 2.46‐3.44).^6^ However, another meta‐analysis of observational studies in post‐ACS patients suggested that transfusion had a neutral or beneficial effect when undertaken at hemoglobin levels <8 g/dL, whereas it could be harmful at hemoglobin >11 g/dL. These apparently contradictory observations are likely related to the limitations of observational studies. The majority of patients undergoing blood transfusion cannot be matched with nontransfused patients due to their markedly different clinical profiles,^37^ indicating that observational studies cannot reliably establish the benefits or risks of transfusion because they are hopelessly influenced by selection bias. In addition, in an observational setting, it is difficult to unravel the effect of anemia, bleeding, and transfusion on outcomes.^1^ There may also be a differential effect of transfusion according to hemoglobin nadir or the presence of overt bleeding.^38^

The two randomized trials that compared restrictive vs liberal transfusion strategies in patients with ACS were small and underpowered.^7^, ^8^ The CRIT randomized pilot study^7^ randomized 45 patients with AMI and hematocrit ≤30% to a liberal vs a conservative transfusion strategy. The primary composite outcome (in‐hospital death, recurrent myocardial infarction, or new or worsening heart failure) occurred in eight patients (38%) in the liberal arm vs three (13%) in the conservative arm (*P* = .046). Conversely, another pilot trial^8^ included patients with ACS or stable angina and hemoglobin <10 g/dL. In that study, the primary outcome (death, myocardial infarction, or unscheduled revascularization at day 30) occurred in six patients (10.9%) in the liberal arm vs 14 (25.5%) in the restrictive arm.

As a consequence of the lack of solid evidence, international guidelines are not fully consistent: the European Society of Cardiology guidelines recommend to consider blood transfusion only in case of compromised hemodynamic status or hemoglobin <7 g/dL^11^, ^12^; the US guidelines do not recommend blood transfusion in patients with non‐STEMI with hemoglobin >8 g/dL.^13^ British guidelines recommend to consider a transfusion threshold of 8 g/dL and a hemoglobin target of 8‐10 g/dL for patients with ACS.^39^ However, the American association of blood banks stated that, given the available evidence, they cannot recommend for or against a liberal or restrictive transfusion threshold in patients with ACS.^40^

There is also currently a wide variation in clinical practice, including in France: in an unpublished web poll performed in 2013 among 227 French sites managing AMI patients, 42% of sites declared using a liberal transfusion policy or having a variable strategy according to physician preference, whereas 58% of sites indicated using a restrictive transfusion policy. Therefore, practice is almost evenly split in France with respect to transfusion strategies in the setting of AMI and anemia, suggesting clinical equipoise. This finding highlights the need for adequately powered randomized trials to establish the role of transfusion during AMI.^9^, ^10^ In addition to REALITY, a North American trial is ongoing: the MINT multicenter randomized trial (NCT02981407) is currently recruiting 3500 patients with anemia and AMI, to test the hypothesis that a liberal transfusion strategy reduces the risk of death or nonfatal AMI within 30 days compared with a restrictive strategy.

Transfusion is a costly treatment (RBC units cost more than 200 euros in France, 140 euros in Spain) and blood is a scarce resource. In addition, beyond the questions of clinical safety, efficacy, and RBC unit costs, blood transfusion is associated with an increased use of medical resources and increased length of stay; hence, the costs of RBC transfusion are probably underestimated.^41^ Finally, blood bank organization, shortage of blood, and costs of storage, testing, and delivery remain major issues in the western world, but even more so in low‐ and middle‐income countries.^42^ For those reasons, a testing strategy combining clinical noninferiority and cost‐effectiveness superiority is legitimate, as establishing clinical noninferiority of a restrictive strategy but reduced costs would be sufficient to establish dominance of this strategy and change clinical practice.

The choice of an open‐label design was justified by the difficulty in blinding the intervention (blood transfusion in the context of AMI). However, the primary clinical effectiveness outcome is a composite of objective clinical outcomes (death and nonfatal major cardiac events: stroke, AMI, and emergency coronary revascularization prompted by myocardial ischemia), which are adjudicated by a CEC blinded to randomization and hemoglobin levels, providing reassurance that these should not be affected by investigator bias.

## 4 CONCLUSION

REALITY is the largest randomized trial to date comparing transfusion strategies in patients with AMI. The trial will provide important clinical and cost‐effectiveness information to guide the management of patients with AMI and anemia.

## CONFLICT OF INTEREST

Dr Ducrocq reports speaker's and/or consulting fees from Amgen, Astra Zeneca, Bayer, BMS, Janssen, Sanofi, and Terumo; is a member of the CEC for Sanofi and Novo Nordisk; and has received travel fees from Astra Zeneca, Biotronik, Bayer, and BMS.

Dr Steg reports grants and nonfinancial support (cochair of the ODYSSEY OUTCOMES trial; as such, he received no personal fees, but his institution has received funding for the time he has devoted to trial coordination, and he has received support for travel related to trial meetings) from Sanofi; research grants and personal fees from Bayer (Steering Committee MARINER, grant for epidemiological study), Merck (speaker fees, grant for epidemiological studies), Sanofi (cochair of the ODYSSEY OUTCOMES trial; cochair of the SCORED trial; consulting, speaking), Servier (Chair of the CLARIFY registry; grant for epidemiological research), and Amarin (executive steering committee for the REDUCE‐IT trial [Disease Reduction of Cardiovascular Events With Icosapent Ethyl‐Intervention Trial]; consulting); and personal fees from Amgen, Bristol‐Myers Squibb, Boehringer Ingelheim, Pfizer, Novartis, Regeneron Pharmaceuticals, Lilly, and AstraZeneca. Dr Steg also has a European application number/patent number, issued on 26 October 2016 (no. 15712241.7), for a method for reducing cardiovascular risk.

## Supporting information


**TABLE S1** Unit costs (URCEco) France (will be adjusted to latest values and severity levels)
**Table S2** Unit costs for Spain
