# Supplementary material for: Restrictive vs liberal red blood cell transfusion strategies in patients with acute myocardial infarction and anemia: Rationale and design of the REALITY trial
Source: Clin Cardiol. 2021 Jan 6;44(2):143–50. doi: 10.1002/clc.23453 (PMC7852166; doi:10.1002/clc.23453)
Supplement: Supplementary file 1 — TABLE S1 Unit costs (URCEco) France (will be adjusted to latest values and severity levels) Table S2 Unit costs for Spain [file CLC-44-143-s001.docx]

**Appendix**

**Restrictive versus liberal red blood cell transfusion strategies in patients with acute myocardial infarction and anemia: Rationale and design of the REALITY trial**

Gregory Ducrocq MD et al.

**Contents**

[REALITY Committees and investigators 2](#_Toc47087497)

[TABLE S1 Unit costs (URCEco) France (will be adjusted to latest values and severity levels) 5](#_Toc47087498)

[TABLE S2 Unit costs for Spain 6](#_Toc47087499)

REALITY Committees and investigators

**Steering committee**

**Chair:** Philippe Gabriel Steg

**Members:** Gonzalo Calvo, Nicolas Danchin, Isabelle Durand-Zaleski, Jose R. Gonzalez- Juanatey, Gilles Lemesle, Manuel Martinez Selles, Cristina Avendano-Sola, Etienne Puymirat, Johanne Silvain, Tabassome Simon, Eric Vicaut

**Scientific coordinator:** Tabassome Simon

**French principal investigator:** Gregory Ducrocq

**Spanish principal investigators:** Gonzalo Calvo, then Jose R. Gonzalez-Juanatey

**Sites and local principal investigators**

**France**

Hôpital Européen Georges Pompidou, Paris (Etienne Puymirat)

CHRU de Lille, Lille (Gilles Lemesle)

Hôpital Bichat Claude Bernard, Paris (Gregory Ducrocq)

Hôpital Pitié Salpêtrière, Paris (Johanne Silvain)

CHU Caremeau, Nimes (Benoît Lattuca)

CHU de Nice Hôpital Pasteur, Nice (Emile Ferrari)

CHU A. Michallon, Grenoble (Gerald Vanzetto)

Hôpital Haut Levêque, Pessac (Pierre Coste)

CHU Dijon, Dijon (Yves Cottin)

CHU Rangueil, Toulouse (Thibault Lhermusier)

Hôpital Lariboisière, Paris (Jean-Guillaume Dillinger)

CH Simone Veil, Eaubonne (Véronique Dormagen)

CHU Jean Minjoz, Besançon (François Schiele)

Hôpital Louis Pradel, Bron (Thomas Bochaton)

CH de Versailles, Le Chesnay (Raphaële Convers-Domart)

Site Sud du CHU d’Amiens Picardie, Amiens (Geneviève Jarry)

Hôpital Ambroise Paré, Boulogne Billancourt (Carma Karam)

Hôpital Trousseau, Tours (Denis Angoulvant)

GH de la Rochelle, La Rochelle (Yann Valy)

CH Annecy Genevois, Pringy (Benjamin Richard)

Centre Hospitalier de Chalon sur Saône (Maxime Fayard)

CH François Mitterrand, Pau (Nicolas Delarche)

CHU La Cavale Blanche, Brest (Martine Gilard)

CH La Timone, Marseille (Thomas Cuisset)

CH d’Angers, Angers (Alain Furber)

Clinique du Diaconat, Mulhouse (Omar Ider)

**Spain**

Hospital Clinic of Barcelona, Barcelona (Joan Albert Arnaiz)

HGU Gregorio Marañón, Madrid (Manuel Martínez-Sellés)

HU de Bellvitge, Barcelona (Albert Ariza)

HCU de Santiago Santiago de Compostella (Jose R. Gonzalez-Juanatey)

Hospital Germans Trias i Pujol, Badalona (Cosme Garcia)

HC de Salamanca, Salamanca (Elisabete Alzola)

HC San Carlos, Madrid (Ana Viana-Tejedor)

HU La Paz, Madrid (Esteban Lopez de Sa)

Hospital Universitario San Juan, San Joan d’Alacant (Alberto Cordero)

**Clinical Events Committee**

**Chair:** Etienne Puymirat (Hôpital Européen Georges Pompidou, Paris)

**Members:** Gregory Ducrocq (Hôpital Bichat, Paris), Emmanuel Sorbets (Hôtel Dieu, Paris), Batric Popovic (CHU Nancy), Jean Guillaume Dillinger (CHU Lariboisière, Paris), Gilles Lemesle (CHU de Lille, Lille), Belén Álvarez (Hospital Universitario de Santiago), Ander Regueiro (Hospital Clínic de Barcelona), Oriol Alegre Canals (Hospital Universitario de Bellvitge)

**Medico-economic analysis**

Isabelle Durand Zaleski, Jérome Frenkiel, Maroua Mimouni, Kevin Zarca (ECEVE, UMRS 1123, URCEco Ile de France, Hôpital de l’Hôtel Dieu); Belén Ruiz-Antoran, Javier Soto (Hospital Puerta de Hierro, Grupo de investigación en Farmacología Clínica, Madrid, Spain)

**Biostatistics/Methodology**

Eric Vicaut (URC Lariboisière, Paris); Alexandra Rousseau, Marine Cachanado (URCEST, Paris)

**Administrative sponsorship and coordination (France):** Assistance Publique-Hôpitaux de Paris, Paris, France: Sandra Paco, Karine Goude-Ory (project leader Sponsor), Elodie Drouet (project leader)

**Coordinating team:** Salima Ammar, Sarah Fedele, Carma Karam, Cynthia Dumoulin, Dominique Damas, Véronique Pignot, Amina Rasnaama, Marie-Lyne Bazerji, Amal Abderrahim, Elise Corsetti, Valentin Charrier, Linghwei Tchon, Alexandra Huttin, Soumaya Boudaya, Julie Dudal, Isabelle Peigney, Vanessa Calle, Meriem Sid, Florian Prever, Céline Chapel, Miassa Bentifraouine, Johanna Canovas, Blandine Le Garrec, Amina Merad, Sara Keffi

**Administrative sponsorship and coordination (Spain):** Sociedad Espanola de Farmacologia Clinica: Pr Cristina Avendano-Sola; Servicio Farmacologia Clinica, Hospital Universitario Puerta de Hierro-Majadahonda, Madrid: Rosa Munoz, Judit Pich, Leticia Pereira Gomez

TABLE S1 Unit costs (URCEco) France (will be adjusted to latest values and severity levels)

| Type of service/ product | Unit cost (€) | Source |
| --- | --- | --- |
| Packed red blood cells | 201.23 per pack | National blood bank* |
| Fresh frozen plasma | 133.41 per unit | National blood bank* |
| Platelets | 238.13 per unit | National blood bank* |
| Cardiac cath | 480 | Scansante† |
| PCI | 1,154 | Scansante† |
| Stent (bare-metal stent) | 488 | Included in the DRG |
| Stent (drug-eluting stent) | 1,000 (range 395−1,363) | List price |
| Coronary artery bypass graft | 16,003 | Scansante† |
| Intensive care unit day (supplement) | 648.84 | Scansante‡ |
| Coronary care unit day (supplement) | 324.42 | Scansante‡ |
| Cardiology ward day | 544.00 | Scansante† |
| REA (resuscitation supplement) | 804.01 | Scansante‡ |
| Intermediate care (telemetry supplement) | 259.54 | Scansante‡ |

*<https://www.legifrance.gouv.fr/affichTexte.do?cidTexte=JORFTEXT000021961438&dateTexte=20181231>

†The per diem costs were calculated for each disease-related group based upon the national cost study. The relevant DRGs were:

| Myocardial infarction without stent by severity level | | Total cost for the DRG € |
| --- | --- | --- |
| 05M041 | Acute myocardial infarction, level 1 | 3,014 |
| 05M042 | Acute myocardial infarction, level 2 | 4,662 |
| 05M043 | Acute myocardial infarction, level 3 | 6,927 |
| 05M044 | Acute myocardial infarction, level 4 | 14,116 |
| Myocardial infarction with stent, by severity level | | |
| 05K051 | Myocardial infarction with stent, level 1 | 4,975 |
| 05K052 | Myocardial infarction with stent, level 2 | 6,822 |
| 05K053 | Myocardial infarction with stent, level 3 | 10,033 |
| 05K054 | Myocardial infarction with stent, level 4 | 18,851 |

‡<https://www.lespmsi.com/tarifs-des-supplements-mco-2018-oqn-et-dgf/>

TABLE S2 Unit costs for Spain

| Cost | Euros |
| --- | --- |
| Cost per intensive care bed day (ICU) | 1,284.15 |
| Cost per coronary care unit bed day (CCU) | 1,053.00 |
| Cost of a hospital ward day | 549.79 |
| Cost of percutaneous coronary intervention | 3,883.00 |

| GRD code | GRD description | Results based on version 32 of APR-GRD |
| --- | --- | --- |
| 174-1 | Percutaneous cardiovascular procedures with AMI – NS1 | 7,260.79 |
| 174-2 | Percutaneous cardiovascular procedures with AMI – NS2 | 8,051.81 |
| 174-3 | Percutaneous cardiovascular procedures with AMI – NS3 | 10,054.06 |
| 174-4 | Percutaneous cardiovascular procedures with AMI – NS4 | 15,325.97 |
| 190-1 | AMI – NS1 | 3,452.17 |
| 190-2 | AMI – NS2 | 3,818.60 |
| 190-3 | AMI – NS3 | 5,246.65 |
| 190-4 | AMI – NS4 | 6,848.45 |

| E 03.1.6.1 | Blood products | Euros |
| --- | --- | --- |
| E 03.1.6.1.01 | Total blood to transfuse | 95 |
| E 03.1.6.1.01.1 | Total blood CPD − ADENINA | 110 |
| E 03.1.6.1.01.2 | Red cell concentration CPD − ADENINA | 140 |
| E 03.1.6.1.02 | Autotransfusion | 124 |
| E 03.1.6.1.03 | Filtered red blood cell concentrate | 124 |
| E 03.1.6.1.04 | Filtered platelet pool | 350 |
| E 03.1.6.1.05 | Apheresis platelet concentrate | 400 |
| E 03.1.6.1.05.1 | Inactivated platelet pool | 350 |
| E 03.1.6.1.05.2 | Inactivated apheresis platelet concentrate | 400 |
| E 03.1.6.1.05.3 | Frozen platelet concentrate | 450 |
| E 03.1.6.1.05.4 | Buffy-coat unit | 15 |
| E 03.1.6.1.06 | Fresh frozen plasma inactivated with methylene blue | 50 |
| E 03.1.6.1.07 | Fresh frozen plasma quarantined | 50 |
| E 03.1.6.1.07.01 | Fresh IgA-deficient plasma | 70 |
| E 03.1.6.1.08 | Umbilical cord blood unit | 21,000 |
| E 03.1.6.1.08.1 | Umbilical cord blood unit for research | 20 |
